# Occurrence of Aflatoxins and Ochratoxin A during Merkén Pepper Powder Production in Chile

**DOI:** 10.3390/foods11233843

**Published:** 2022-11-28

**Authors:** Jéssica Costa, Carla Santos, Célia Soares, Rodrigo Rodríguez, Nelson Lima, Cledir Santos

**Affiliations:** 1 Programa de Doctorado en Ciencias de Recursos Naturales, Universidad de La Frontera, Temuco 4811-230, Chile; 2 Laboratório de Cultura de Tecidos Vegetais, Departamento de Biologia, Instituto de Ciências Biológicas-ICB, Universidade Federal do Amazonas, Av. Rodrigo Otávio Jordão Ramos 3000, Bloco E, Setor Sul, Manaus 69077-000, AM, Brazil; 3 CEB-Centre of Biological Engineering, Campus de Gualtar, Micoteca da Universidade do Minho (MUM), University of Minho, 4710-057 Braga, Portugal; 4 LABBELS (Associate Laboratory, Braga/Guimarães), Campus de Gualtar, University of Minho, 4710-057 Braga, Portugal; 5 Department of Chemical Science and Natural Resources, Universidad de La Frontera, Temuco 4811-230, Chile

**Keywords:** aflatoxin B1, Merkén, ochratoxin A, pepper powder, post-harvest, spoilage fungi

## Abstract

Berry fruits of *Capsicum annuum* L. cv. “Cacho de Cabra” are used for the manufacture of a traditional pepper powder known as Merkén. In the present study, aflatoxins (AFs) and ochratoxin A (OTA) contamination in berry fruits of *C. annuum* was determined at harvest, drying, and smoking stages of Merkén production, in cumin and coriander seeds used as Merkén ingredients, and in the final packaged Merkén produced by local farmers. Additionally, Merkén samples from local markets in the region of La Araucanía (Chile) were also evaluated. Chromatographic analysis was based on a qualitative method. AFs and OTA were not detected on pepper pods and seeds. There was no detection of AFs and OTA on cultured *Aspergillus* and *Penicillium* strains isolated from pepper pods, cumin and coriander seeds and Merkén. The lack of AFs/OTA-producers among the isolated fungal species can explain and support the absence of contamination in pepper pods. In contrast, the AFB_1_ was detected in 75% of Merkén obtained from farmers and 46% of Merkén samples purchased from local markets; while OTA was detected in 100% of Merkén samples obtained from farmers and local markets. In the Merkén production chain, the harvest and post-harvest are key stages for fungal growth while the commercialization stage is highly susceptible to AFs and OTA contamination.

## 1. Introduction

Merkén is a ground-up pepper seasoning originally produced by Mapuche communities, an Amerindian community mainly living in south-central Chile and south-west Argentina [1,2]. The artisanal smoked manufacturing process and the use of Chilean-specific raw materials (e.g., *Capsicum annuum* L. cv. “Cacho de Cabra”, coriander and cumin seeds) are the main reason behind the distinguishing organoleptic characteristics of Merkén (e.g., color, flavor, and aroma) [1,3].

Due to its exotic flavor, Merkén is widely accepted in both Chilean and overseas markets, which has been boosting the development of local start-ups. Overall, these initiatives seek to reach the traditional and gourmet segment, preserving the Mapuche agri-food culture [4]. On a large production scale, the four leading brands in the condiment market in Chile include Merkén and pepper derivative products in their condiment range [5,6].

Merkén production begins with the collection of *C. annuum* L. cv. “Cacho de Cabra”, a pepper ecotype of the region of Araucanía, Chile. Thereafter, pepper pods are dried by sun exposure or inside “Rukas”, which are typical Mapuches’ houses made of wood and straw [1,2]. Then, farmers smoke the fruits for approximately half an hour on a native wood fire. The last stage is grinding pepper pods and mixing them with other ingredients. The final Merkén composition has about 70% of pepper, 20% of both coriander and cumin seeds, and 10% of sodium chloride [5].

Merkén’s quality is directly related to the quality of its raw ingredients but also the careful management of good agricultural practices (GAP) and good handling practices (GHP) during its production chain [7]. In Chile, Merkén manufacture is predominantly carried out following traditional methods [1]. Overall, post-harvest pepper pods are affected by changes in temperature, exposure to dust, wind, and insect infestation [8]. Further processing such as storage, packing, and transportation also affects the low-standard control of the raw materials, which can lead to fungal growth [5,8,9,10].

Several studies reported that pepper pods from different varieties of *Capsicum* are highly susceptible to the proliferation of spoilage genera fungi such as *Botrytis*, *Cladosporium*, *Harzia*, *Rhizopus*, and *Phytophthora*, including yet mycotoxigenic species such as *Aspergillus flavus*, *A. niger*, *A. ochraceus*, *Penicillium expansum*, and *P. thomii* [9,11,12]. Overall, pepper pods from field and post-harvest are predominantly contaminated with fungal strains of *Alternaria*/*Fusarium* and *Aspergillus*/*Penicillium*, respectively [11,13].

Mycotoxins such as aflatoxins (AFs) and ochratoxin A (OTA) are among the most important contaminants in *Capsicum* from a consumer point of view [13]. Moreover, Zearalenone (ZEN), Fumonisins (FB), Trichothecenes, and *Alternaria* toxins were also widely found in *Capsicum* [13,14].

Between 2020 and 2021, 27 cases of contamination of imported *Capsicum* products were reported by the European Rapid Alert System for Food and Feed (RASFF) [15]. Of those, 18 were classified as rejections at the border, 6 as alerts, and 3 as information notifications. According to the RASFF, 21 notifications referred to AFs and 6 to OTA. In Chile, total AFs (range from <limit of detection (LOD) to 14.12 μg/kg) and OTA (range from <LOD to 416.3 μg/kg) were detected in samples of pepper pods and Merkén [16].

The protracted consumption of foods contaminated with aflatoxins (B_1_, B_2_, G_1_, and G_2_) and OTA is a serious risk to human health. AFB_1_, AFB_2_, AFG_1_, and AFG_2_ are carcinogenic compounds to humans, while OTA is potentially carcinogenic [17]. These mycotoxins can affect the immune system and injure different target organs (e.g., kidneys, liver, gallbladder) [18,19,20]. In Chile, the regulation for mycotoxin in spices, including *Capsicum*, establishes the maximum tolerance levels (MTL) for total AFs at 10 µg/kg. However, the same legislation does not regulate the MTL for OTA in spices [21].

AFs and OTA contaminations have widely been reported at different points of the pepper production chain (e.g., harvest, dried, smoking, sale, etc.) [14,22]. Mycotoxins are resistant to food processing and can remain throughout the food chain from “farm to fork”, emphasizing the need for sample evaluation from “pepper pod to final Merkén” [23]. Despite the best effort to guarantee the food safety of Chilean pepper products, mycotoxin analysis in Chile has been mainly carried out in samples obtained from supermarkets [21]. Up to now, there is no information available about critical points of mycotoxin contamination over different stages of the Merkén production chain [8]. In addition, to the best of our knowledge, no information about mycotoxin contamination in Merkén commercialized in local Chilean markets is available in the literature.

This study aimed to determine AFs (B_1_, B_2_, G_1_, and G_2_) and OTA occurrence in berry fruits of *C. annuum* L. cv. “Cacho de Cabra” during manufacturing (harvest, drying, and smoking stages), in added ingredients (cumin and coriander seeds), in the final packaged Merkén produced by local farmers, and in Merkén commercialized in local markets of the region of La Araucanía, Chile. Furthermore, the aflatoxigenic and ochratoxigenic potential of fungal strains isolated from each substrate were also assessed.

## 2. Materials and Methods

### 2.1. Sampling

**Figure 1:**
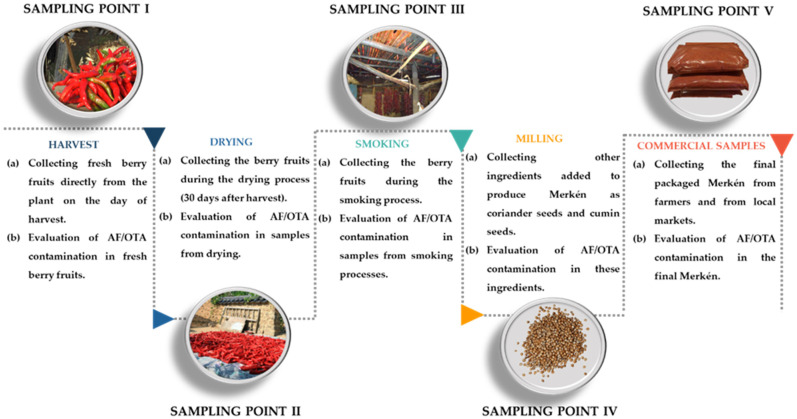
Sampling points throughout the Merkén production chain (a) and mycotoxigenic analysis carried out on samples from each stage (b).

Berry fruits of *C. annuum* L. cv. “Cacho de Cabra”, cumin seeds, coriander seeds, and Merkén were collected from April to June 2017 in the region of La Araucanía, Chile. A flow diagram of sampling during Merkén production is given in detail in Figure 1; samples were collected from all production stages. Berry fruits of *C. annuum* L. cv. “Cacho de Cabra” were provided by 8 farmers as previously reported by Costa et al. [11]. Briefly, samples of pepper pods were collected at 3 different sampling points: (1) on the day of ripe fruits harvest (SP I); (2) during the drying process (1 month after harvest, SP II); and (3) during the smoking process (SP III). For each sampling point, 10 pepper pods were obtained from each farmer, totaling 240 samples collected and analyzed (SP I, *n* = 80; SP II, *n* = 80; SP III, *n* = 80). The samples of seeds of coriander (SP IV, 25 g), cumin (SP IV, 15 g), and final Merkén samples (SP V, *n* = 8) were also provided by the same farmers. In addition, 13 samples of Merkén (SP V) were randomly purchased from the local market (*n* = 13, 100 g).

### 2.2. Mycotoxins Extraction from Substrates

For mycotoxin analysis, the 10 pods obtained from each producer (SP I to SP III) and the coriander and cumin seeds were finely grounded and blended. Each sample was separately stored at 4 °C in plastic bags until analysis.

For AFs/OTA extraction, static sampling of crushed *C. annuum* pods (5 g), seeds of coriander and cumin (5 g), and Merkén (2 g) of each producer were performed. Each sample was mixed with extraction solution (0.2 g of NaCl; 10 mL of methanol: water 8:2; and 5 mL of hexane) in 100 mL Erlenmeyer flasks were shaken on a mechanical shaker at 150 rpm for 1 h at room temperature. Each solution was filtered through Whatman^TM^ N^o^ 4 filter paper (Maidstone, UK) and separated using a funnel. After phase separation, 5 mL of the aqueous layer was diluted with 30.7 mL of PBS buffer and filtered using glass microfiber Whatman^TM^ filters.

For extract cleanup, 10 mL of each solution was added to an AflaOchra HPLC immunoaffinity column (VICAM column, Watertown, NY, USA) at a flow rate of mL min^−1^. The column was washed with 10 mL of PBS buffer at a flow rate of 2 mL min^−1^ and the mycotoxins were eluted with 1.5 mL of methanol. The eluted samples were analyzed by HPLC as described in Section 2.5.

### 2.3. Mycological Analyses

The mycological assessment was performed throughout each point in the Merkén production chain (Figure 1). Mycobiota isolation of *C. annuum* pods from SP I, SP II, and SP III was previously reported in Costa et al. [11].

For Merkén samples, fungal strain isolation was performed following the dilution method described by Samson et al. [24]. Ten grams of each sample was homogenized in 90 mL of peptone water solution. Serial dilutions were made and 0.1 mL aliquots were inoculated in duplicate onto Malt Extract Agar (MEA, malt extract 20 g L^−1^, mycological peptone 1 g L^−1^, agar 20 g L^−1^, glucose 20 g L^−1^), Dichloran Rose Bengal Chloramphenicol Agar (DRBC, KH_2_PO_4_ 1 g L^−1^, MgSO_4_·7H_2_O 0.5 g L^−1^, peptone 5 g L^−1^, dichloran 0.002 g L^−1^, chloramphenicol 0.1 g L^−1^, agar 15 g L^−1^, glucose 10 g L^−1^, rose bengal 0.025 g L^−1^), and Dichloran 18% Glycerol Agar (DG18, mycological peptone 5 g L^−1^, glucose 10 g L^−1^; KH_2_PO_4_ 1 g L^−1^, MgSO_4_·7H_2_O 0.5 g L^−1^, glycerol 220 g L^−1^, dichloran 0.002 g L^−1^, chloramphenicol 0.1 g L^−1^, agar 15 g L^−1^) media.

Fungal isolation from coriander and cumin seeds was carried out based on the agar plate method [25]. Five seeds of coriander or cumin were equally placed on MEA, DRBC, and DG18 media plates. Ten replicates were performed for each sample.

All plates were incubated in the dark at 25 °C for 7 days. After the incubation period, all colonies of potentially mycotoxigenic genera were transferred for sub-culturing to plates of MEA and Potato Dextrose Agar (PDA, 200 g L^−1^ of infusion from potatoes, glucose 20 g L^−1^, agar 15 g L^−1^). Taxonomic identification of fungal strains at the genus level was performed according to macro-and micro-morphological traits with appropriate keys [26,27,28]. All the 31 fungal strains isolated in the present study were deposited at the Banco de Recursos Microbiológicos BRmB-UFRO (Faculty of Engineering and Science, Universidad de La Frontera, Temuco, Chile), at the Culture Collection CCCT/UFRO (Universidad de La Frontera, Temuco, Chile), and at the Micoteca da Universidade do Minho (MUM, University of Minho, Braga, Portugal).

#### 2.3.1. Genomic DNA Extraction

The genomic DNA of each isolate was extracted using a modified protocol described by Rodrigues et al. [29] and detailed in Costa et al. [11].

#### 2.3.2. PCR Amplification

To identify the fungal strains, partial amplification of the internal transcribed spacer of the ribosomal DNA (ITS) region or β-tubulin gene (*benA*) was performed. ITS is regarded as the universal barcode for fungal identification [30]. Nevertheless, for specific genera such as *Penicillium* and *Aspergillus*, *benA* is the more informative primary barcode when trying to achieve species-level identification [31,32].

ITS was amplified using primers ITS1 (5′-TCC GTA GGT GAA CCT GCG G-3′) and ITS4 (5′-TCC TCC GCT TAT TGA TAT GCC-3′) designed by White et al. [33]. *BenA* was amplified using primers Bt2a (5′-GGT AAC CAA ATC GGT GCT GCT TTC-3′) and Bt2b (5′-ACC CTC AGT GTA GTG ACC CTT GGC-3′) designed by Glass and Donaldson [34]. For both regions, PCR reactions included 25 μL Taq DNA polymerase Master Mix 2× (VWR Life Science, Leuven, Belgium), 1 μL of each primer at 10 mM, and 2 μL of genomic DNA in a final volume of 50 μL. PCR parameters used in the thermal cycler for *benA* and ITS were: 95 °C for 5 min, 35 cycles of 95 °C for 1 min, 56 °C for 45 s, 72 °C for 90 s, and a final extension at 72 °C for 10 min.

Amplification success was verified on 1% (*w*/*v*) agarose gels and PCR products purified using NZYGelpure kit (NZYTech Lda, Lisbon, Portugal) according to the manufacturer’s instructions and sent for Sanger sequencing to StabVida (Madan Parque, Caparica, Portugal). To provide species identification, phylogenetic analyses were performed as described in Costa et al. [11] against sequences of reference species retrieved from the NCBI database.

### 2.4. Toxigenic Capacity of Fungal Isolates

Potential mycotoxigenic fungi strains were tested for the production of AFs and OTA. All *Aspergillus* and *Penicillium* strains isolated from SP I, SP II, and SP III were tested for AFs production. These strains were previously assessed for OTA production on a culture medium [11]. *Aspergillus* and *Penicillium* strains isolated from coriander and cumin seeds and Merkén samples were tested for AFs and OTA production. Each fungal strain was placed onto 6 cm diameter plates with Czapek yeast extract agar medium (CYA, agar 15 g L^−1^, K_2_HPO_4_ 1 g L^−1^, sucrose 30 g L^−1^, yeast extract 5 g L^−1^, 10 mL of Czapek concentrate, 1 mL of trace metal solution) and grown for 10 days at 25 °C. For AFs/OTA extraction, three agar plugs were removed from one colony and placed into a 4 mL vial, where 2 mL of methanol was added. After 1 h, the extract was filtered through 0.2 μm syringe filters (Filter-Bio, Nantong, China) and analyzed by HPLC [35].

### 2.5. Mycotoxin Detection

Overall, chromatographic analysis was based on a qualitative method. Samples were taken as positive, for each of the analyzed mycotoxins, when yielding a peak at a retention time similar to each standard. Each sample was scored by the presence/absence of the analyzed mycotoxins, being classified as not detected (−) and detected (+) [29].

For AFs detection, samples were analyzed using High-Performance Liquid Chromatography (HPLC Waters, Milford, MA, USA) equipped with a Jasco FP-920 fluorescence detector (365 nm excitation wavelength; 435 nm emission wavelength), using a photochemical post-column derivatization reactor (PHRED unit—Aura Industries, San Diego, CA, USA). Chromatographic separation was performed on a reverse phase C18 column (Waters Spherisorb ODS2, 4.6 mm × 250 mm, 5 µm), fitted with a pre-column with the same stationary phase. The mobile phase used was pumped at 1.0 mL/min and consisted of an isocratic mobile phase as follows: water: acetonitrile: methanol (3:1:1, *v*/*v*).

For total AFs, retention times were 11 min for AFG_2_, 12.90 min for AFG_1_, 14.39 min for AFB_2_, and 17.18 min for AFB_1_. AFs determination in samples was based on a method using a total AFs concentration range from 0.05 to 50 ppb. Samples were taken as positive for each of the toxins when peaks were at a retention time similar to each standard, with an area height five times higher than the baseline noise.

OTA analysis was performed as described in Costa et al. [11]. OTA was identified by comparison of the peak samples’ retention time with that of the standards. Standards were prepared by serially diluting a primary OTA stock solution (25 μg/mL). Chromatograms with OTA concentration ranging from 0.05 to 50 ppb were obtained. Samples were taken as positive for each of the toxins when peaks were at a retention time similar to each standard, with a peak height five times higher than the baseline noise.

## 3. Results and Discussion

### 3.1. Fungal Contamination

**Figure 2:**
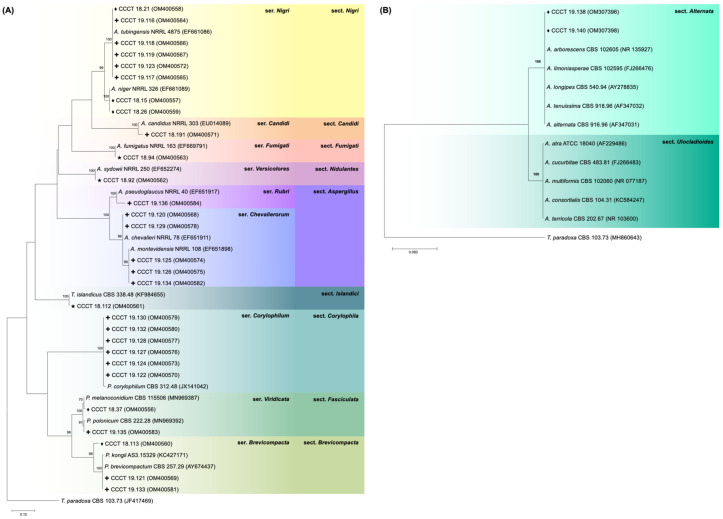
Phylogenetic analyses of *benA* and ITS sequence data of the 31 strains isolated from SP IV (coriander ♦, cumin ★) and SP V (Merkén **+**) samples collected in the region of La Araucanía, Chile. GenBank accession codes are indicated in parenthesis. *Trichocoma paradoxa* CBS 103.73 was used as an outgroup. The percentage of trees in which the associated taxa cluster together in the bootstrap test (1000 replicates) is shown above the branches. The trees are drawn to scale with branch lengths measured in the number of substitutions per site. All positions with less than 95% site coverage were eliminated. (**A**) *benA* phylogenetic tree including *Aspergillus*, *Talaromyces*, and *Penicillium* strains. Selected model: K2 + G + I. The final dataset included 44 nucleotide sequences and a total of 251 positions. (**B**) ITS phylogenetic tree of *Alternaria* strains. Selected model: K2. The final dataset included 13 nucleotide sequences and a total of 426 positions.

Merkén is a substrate with a low water activity (a_w_) and high NaCl concentration. Both intrinsic factors narrow growth conditions, favoring a specific fungi subset. The results presented here show that *Aspergillus* and *Penicillium* were the dominant genera isolated from Merkén samples (Table 1; Figure 2). The findings obtained in the present study are in accordance with those reported by Chuaysrinule et al. [36], Costa et al. [11], and Santos et al. [13], in which *Aspergillus* and *Penicillium* are the dominant fungal genera in pepper powder and also in Merkén raw material (e.g., dried and smoked *C. annuum* pods).

*Penicillium brevicompactum* was the only species isolated from all sampling points of the Merkén production chain, which means fresh, dried, smoked fruits of *Capsicum*, and Merkén. This suggests that this species is well adapted to this particular crop and derived product. The other species of *Aspergillus* (*A. candidus*, *A. chevalieri*, *A. fumigatus*, *A. pseudoglaucus*, *A. tubingensis*) and *Penicillium* (*P. corylophilum* and *P. polonicum*) isolated from Merkén have also been found in other pepper-derivative products [9,11,12]. These isolates encompass the xerophilic/xerotolerant and osmotolerant species, which can survive repeated desiccation–rehydration cycles [37,38]. In addition, cumin and coriander seeds that are added to the Merkén mixture can be supplementary entry points for fungal contamination.

The obtained mycobiota profile fits the requirements needed to overcome suboptimal conditions found in Merkén. The origin of the *Capsicum* fruits, the hygiene conditions during storage and transport, the water activity in Merkén, and its raw material are key factors to control fungal proliferation [7,10].

### 3.2. Occurrence of AFs and OTA

Under the conditions analyzed in the present study, AFs and OTA were not detected in berry fruit samples of *C. annuum* L. cv. “Cacho de Cabra” from harvest (SP I), drying (SP II), and smoking (SP III) stages. The absence of AFs and OTA in pepper pods produced by these farmers does not guarantee that *C. annuum* products such as Merkén are free of mycotoxins. Conversely to the results presented here, several studies widely reported mycotoxin contamination in *Capsicum* pods [22,39], pepper powder [40], and pepper-based products [14,41]. Ikoma et al. [42] analyzed dried peppers from Chile, showing a contamination range from 163.4 to 1059.2 μg/kg with an average OTA amount of 355.6 μg/kg. Similarly, the Chilean Mycotoxin Surveillance Program, which analyzed mycotoxin contamination in Chilean food samples between 2013 and 2017, reported that OTA was detected in 81 samples of Merkén and *Capsicum* pods ranging from <LOD to 416.3 μg/kg; and total Afs were detected in 45 samples of Merkén and *Capsicum* pods ranging from <LOD to 14.12 μg/kg [16]. In all cases, there is no information regarding the agricultural practices used in the production of the analyzed pepper pods.

Regarding coriander and cumin seeds, both ingredients are added to Merkén during the milling step (SP IV) and are pointed out as a possible route of mycotoxigenic contamination to Merkén. However, in the present study, AFs and OTA were not detected in the analyzed seed samples.

The occurrence of AFs and OTA was also analyzed in Merkén samples provided by farmers (SP V; *n* = 8) and purchased from local markets (SP V; *n* = 13) in the region of La Araucanía (Chile) (Table 2). AFB_1_ was detected in 75% of the Merkén samples obtained from farmers and 46% of the Merkén samples purchased from local markets. AFB_2_, AFG_1_, and AFG_2_ were not detected in any sample evaluated in the present study. OTA was detected in 100% of Merkén samples obtained from farmers and local markets. The protracted consumption of food contaminated with both toxins is a risk factor for human health. According to Foerster et al. [43], consumers of high amounts of Merkén and *Capsicum* pepper could be at risk of mycotoxin exposure. Further, the analyses of urine samples from a fraction of the Chilean rural population revealed a significant correlation between AFB_1_ and *Capsicum* powder consumption (*R*^2^: 0.18, *p* = 0.03) [44]. AFB_1_ exposure through *Capsicum* and derivatives intake has been suggested as one of the risk factors responsible for high rates of gallbladder cancer among Chilean women [19,42,45,46,47,48]. In the present study, the co-occurrence of AFB_1_ and OTA was detected for c.a. 57% of Merkén samples (12 out of 21 samples). Mycotoxin co-occurrence in pepper powder is widely reported [14,39,49]. Santos et al. [50] evaluated the co-occurrence of AFs, OTA, and ZEN in pepper samples commercialized in Spain. According to the authors, 65% of the pepper samples contained more than one mycotoxin. Similarly, Ozbey and Kabak [40] reported the co-occurrence of AFs and OTA in c.a. 41% of the analyzed red pepper powder samples.

Chilean companies that trade Merkén and derivative products of *Capsicum* have sought to enhance and solidify their presence in the international market, especially in Europe and the United States of America. Thus, guaranteeing food safety is a key point for the expansion of these business trades. Although it is a risk factor, up to now Chilean legislation does not regulate the maximum limits of OTA in spices. In Chile, the regulation for mycotoxins in spices, including *Capsicum*, establishes MTL at 10 µg/kg, only for total AFs. Conversely, the European Commission (EC) established rigorous legislation for mycotoxin in food and feed for all countries of the European Union. The regulations for AFs in *Capsicum* fruits with MTL are set at 10 µg/kg for total AFs (AFB1 + AFB2 + AFG1 + AFG2) and at 5.0 µg/kg for AFB1 [51]. The regulation was recently updated with the maximum levels of OTA in spices of 20 µg/kg for *Capsicum* powder and 15 µg/kg for mixtures of *Capsicum* with other species [52]. No maximum tolerable *Fusarium*-produced mycotoxins or patulin concentration has been established for pepper powder to date. Combined intake of different types of mycotoxins may lead to a synergistic or at least additive effect [6,19]. Currently, there is no regulation for combined contamination of mycotoxins in different foods, except for the control of the four aflatoxins (AFB_1_, AFB_2_, AFG_1_, and AFG_2_) together [53].

The ability of the *Penicillium* (*n* = 113) and *Aspergillus* (*n* = 35) isolates from SP I, SP II, and SP III to produce aflatoxins was also evaluated in the present study. Under the analyzed conditions, none of the strains isolated from *Capsicum* pods produced AFs. The isolated fungal species reported herein were also previously assessed as non-OTA producers [11]. The lack of AFs and OTA strain producers among the isolated fungal species can explain and support the absence of contamination in berry fruits of *C. annuum* L. cv. “Cacho de Cabra”. Regarding the *Aspergillus* (*n* = 17) and *Penicillium* (*n* = 11) strains isolated from cumin and coriander seeds and Merkén, none of the strains produced AF or OTA above the detection levels. Conversely, OTA (100% of samples) and AFB_1_ (57% of samples) were present in Merkén samples. Once Merkén samples from local markets came from different producers than the ones analyzed here, it was not possible to ensure that *Capsicum* pods used in their production were free of spoilage fungi and mycotoxins. Then, it is possible to suggest that a process of mycotoxin accumulation at the endpoint is occurring, where only the toxin is detected and not the mycotoxigenic fungal strains. Additionally, according to the data obtained in the present study, it was not possible to establish a direct relationship between the isolated mycobiota and the mycotoxins contamination of Merkén. For Merkén samples obtained from the local farmers, mycotoxins accumulation throughout the production stage seems unlikely since no contamination was detected in the used raw material and the isolated fungal strains have not produced toxins. This may suggest that suitable conditions found in Merkén composition, and not in the other subtracts, may have triggered AF/OTA biosynthesis by an indigenous strain or also by an unknown fungal producer of these mycotoxins.

Besides a strain’s toxigenicity, the substrate composition (NaCl, capsaicinoids, capsinoids, etc.) and ecophysiological factors (lower a_w_ level, temperature) can play a key role in the up/downregulation of mycotoxin biosynthesis [54,55]. Concerning Merkén, despite being a pepper-based product, the high NaCl concentration (10% *w*/*w*) makes this substrate an unlikely ecological habitat for the berry fruits of *C. annuum* L. cv. “Cacho de Cabra”. The NaCl-rich environment can promote the OTA biosynthesis by fungi (e.g., *Penicillium nordicum*) as an adaptive strategy to osmotic stressful conditions [56,57,58,59,60]. Ochratoxin A has been previously detected in food substrates rich in salt such as cheese, dry-cured sausages, and, according to the results from this survey, in Merkén. In addition, the antifungal compounds (e.g., capsaicin) present in pepper powder and also *Capsicum* berry fruits can inhibit fungal growth including aflatoxigenic strains by downregulating AF biosynthesis [54]. The role of NaCl and capsaicinoids on fungal community dynamics and mycotoxin biosynthesis in *C. annuum* berry fruits and Merkén is still unclear. Further analysis is required to assess the susceptibility of this substrate to other mycotoxins since fungi strains isolated from berry fruits of *C. annuum* L. cv. “Cacho de Cabra” [11] and Merkén are potentially able to produce citrinin (*P. citrinum* and *P. expansum*), fumonisins (*A. niger*), gliotoxin (*A. fumigatus*), or penicillic acid (*P. cyclopium*, *P. melanoconidium*, *P. polonicum*, and *P. viridicatum*), among other secondary metabolites [14,36].

### 3.3. Recommendations for Merkén Production Chain Food Safety

**Figure 3:**
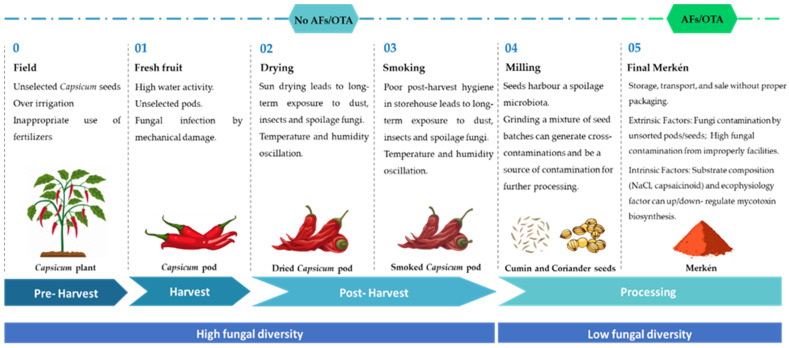
Overview of critical points for contamination with spoilage fungi and AFs/OTA, according to results of the present study. The main factors affecting food safety at each point of the Merkén production chain are highlighted.

Based on the data presented herein, it is possible to infer that harvest (field) and primary processing of post-harvest (drying, smoking, and milling) are not potential critical points for OTA and AFs contamination (Figure 3), even though all these stages are susceptible to fungal growth [11]. However, it is worth pointing out that the mixture of unsorted pods, during the milling process, can lead to cross-contamination [6]. For small farmers, the dynamic between milling and sale may be more straightforward. Overall, Merkén remains on the farm and in store for a shorter period, either for their own consumption or to be passed directly to the final consumer. When passed to cooperatives through a middleman or sold at local markets, Merkén can be held in storage over a longer period [1].

Unlike in previous steps, the endpoint of the Merkén production chain, which corresponds to the commercialization stage, is susceptible to AFs and OTA contamination. At this stage, Merkén is usually sold in bulk, unpackaged (big open bags or wood/plastic boxes). The absence of a physical barrier makes this spice susceptible to surrounding dirtiness and also to airborne spores. In this study, some clinically important fungal pathogens (e.g., *A. chevalieri*, *A. fumigatus*, *A. tubingensis*) were isolated from Merkén samples. Therefore, even mycotoxin-free samples can still be a risk due to their mycological burden [61,62,63]. During storage (after milling) and commercialization, conditions of humidity (≤70%), temperature (≤5 °C), and sanitation of warehouses must be strictly controlled and stabilized; otherwise, it can allow new fungal infections or suitable conditions for the growth of indigenous mycobiota and mycotoxin production [7]. This is an aspect worth considering in Merkén production, mainly because in Chile (southern hemisphere) the harvest and processing of *Capsicum* pods up to Merkén manufacture coincide with autumn, a period in which the temperatures and hours of daylight decrease quickly [64].

In the present work, the detection of AFB_1_ and OTA and the high mycological burden in Merkén samples and its raw materials emphasize the need for improvements in its production chain. Low-cost intervention measures may be successfully applied to reduce exposure to spoilage fungi and mycotoxins in local farming [65]. For example, to avoid soil contact and for easy displacement in case of rain conditions during the drying step, instead of being spread on the floor, *Capsicum* fruits should be spread onto a clean canvas.

The fruits should be well spread over the tarpaulins, without layers, for even drying. The tarpaulins should be washed, if possible, with bleach, then dried in the sun and stored in a clean place; the area used for drying *Capsicum* pods in the sun should be inaccessible to animals (e.g., rodents, birds, insects). For this, a fiberglass screen can be used to fence the space. Thereafter, the warehouse where the smoking of the *Capsicum* pods takes place usually has a central sawdust fire, dirt floor, and is several wood stories high, where the *Capsicum* pods are placed.

The warehouse floor can be concreted to minimize dust contamination and to be easier to wash and clean. The wooden shelves can be covered with natural-fiber mats or tarpaulins, preventing the pods from suffering injuries due to friction with the wood. In the milling stage, the pods can be stored in jute and polyethylene bags, which can be cleaned regularly. *Capsicum* pods and additional ingredients (seeds) must undergo a visual inspection, and any lesion or microbiological contamination should be properly discarded.

The grinder must be cleaned before and after use. Merkén can be stored in new/clean burlap or polypropylene bags. The bags should be stored off the ground, preferably on pallets in a clean and airy environment. In the sales stage, Merkén sold in bulk at local fairs should ideally be stored in airtight containers. Currently, this spice is stored in open jute or canvas bags. The products are exposed and susceptible to dirt, insects, moisture, and fungal contamination. Additionally, it can affect Merkén’s organoleptic properties. Finally, regular Training Programs on Good Agricultural Practices (GAP) and Good Manufacturing Practices (GMP) should be available to rural farmers and stakeholders of *Capsicum* pepper.

## 4. Conclusions

This study analyzed for the first time all points of Merkén production chains regarding mycotoxin contamination. Our results show the absence of AFs and OTA in coriander and cumin seeds and in *Capsicum* pods from all production stages (e.g., harvest, drying, and smoking), pointing out that harvest (field) and initial processing of post-harvest (dried, smoking, and milling) are not critical stages for OTA and AFs contamination. These findings suggest that at least the raw materials (e.g., *C. annuum* pods and seeds of coriander and cumin) of Merkén obtained from farmers are not the main source of AF and OTA contamination. For Merkén samples obtained in local markets, it is not possible to reach the same conclusion since its raw materials were not analyzed.

Merkén commercialization, the last stage of the production chain, is a critical point for AFs and OTA contamination. However, it was not possible to establish a direct relationship between the isolated mycobiota and the AF and OTA contamination observed in Merkén. Isolated *Penicillium* and *Aspergillus* species did not produce any of the analyzed mycotoxins under the used in vitro conditions. However, the unique intrinsic factors (high NaCl concentration, presence of capsaicinoids and capsinoids, and ecophysiological factors) found in Merkén composition in addition to the extrinsic factors (fungi contamination by unsorted pods/seeds, fungal contamination from substandard facilities) can provide suitable conditions for AF/OTA biosynthesis by indigenous strains or also by an unknown producer of these mycotoxins.

From the food safety point of view, the detection of AFB_1_ and OTA and the high mycological burden in Merkén samples emphasize the need for production chain improvements. The application of low-cost corrective actions based on Good Manufacturing Practices (GMP), Good Hygienic practices (GHP), and Good Storage Practices (GSP) should be successfully applied along the Merkén supply chain.

## Figures and Tables

**Table 1:** Mycobiota isolated from Merkén pepper samples and coriander and cumin seeds after 7 days of incubation on DRBC, DG18, and MEA at 25 °C. The number of isolates of each species is indicated in parenthesis. Detailed phylogenetic analyses can be found in Figure 2.

Spices	Fungal Species		
	*Aspergillus* spp.	*Penicillium* spp.	Other Genera
**Merkén**	*A. tubingensis* (5)	*P. brevicompactum* (2)	
	*A. candidus* (1)	*P. corylophilum* (6)	
	*A. chevalieri* (2)	*P. polonicum* (1)	
	*A. pseudoglaucus* (1)		
	*A. montevidensis* (3)		
**Coriander**	*A. niger* (2)	*P. kongii* (1)	*Alternaria* sect. *Alternata* (2)
	*A. tubingensis* (1)	*P. melanoconidium* (1)	
**Cumin**	*A. fumigatus* (1) *A. sydowii* (1)		*T. islandicus* (1)

**Table 2:** AFs and OTA results for Merkén from farmers (*n* = 8) and markets (*n* = 13) of the region of La Araucanía, Chile.

Source	Samples	OTA (µg/kg)	AFB_1_ (µg/kg)
Farmer	I	+	+
	II	+	+
	III	+	+
	IV	+	+
	V	+	+
	VI	+	+
	VII	+	−
	VIII	+	−
Local markets	1	+	−
	2	+	−
	3	+	−
	4	+	+
	5	+	+
	6	+	+
	7	+	−
	8	+	+
	9	+	−
	10	+	+
	11	+	−
	12	+	+
	13	+	−

(+) Detected; (−) Not detected.

## Data Availability

The datasets presented in this study can be found in online repositories. The names of the repository/repositories and accession number(s) can be found below. Beta-tubulin (*benA*) partial sequences: https://www.ncbi.nlm.nih.gov/search/all/?term=, OM400555-OM400584; Internal Transcribed Spacer (ITS) partial sequences: https://www.ncbi.nlm.nih.gov/search/all/?term=, OM307396-OM307401.

## References

[1] Costa J., Lima N., Santos C., Oliveira L.A., Jesus M.A., Jackisch-Matsuura A.B., Gasparotto L., Oliveira L.G.S., Lima-Neto R.G., Rocha L.C. (2019). Chilean pepper: Spoilage fungi and mycotoxins contamination risk in Capsicum products. Conhecimento, Conservação e uso de Fungos.

[2] Muñoz-concha D., Quiñones X., Pablo J. (2020). Chili Pepper Landrace Survival and Family Farmers in Central Chile. Agronomy.

[3] Oyarzún M.T., Riveros H., Vandecandelaere E. (2013). Cómo Promover La Calidad Vinculada al Origen Para Contribuir al Desarrollo en América Latina: Enseñanzas de Cuatro Casos Piloto.

[4] (2021). ProChile. https://acceso.prochile.cl/?s=Merk%C3%A9n.

[5] Fundación para la Innovación Agraria (FIA) (2010). Resultados y Lecciones en Ají Merkén con Alto Valor Agregado.

[6] Costa J., Rodríguez R., Garcia-Cela E., Medina A., Magan N., Lima N., Battilani P., Santos C. (2019). Overview of fungi and mycotoxin contamination in *Capsicum* pepper and in its derivatives. Toxins.

[7] Ozturkoglu-Budak S. (2017). A model for implementation of HACCP system for prevention and control of mycotoxins during the production of red dried chili pepper. Food Sci. Technol..

[8] Di Pillo F., Martínez N. (2018). Ocratoxina A en Ají y merkén, Chile Perfil de Riesgo/ACHIPIA N.°02/2018 Ocratoxina.

[9] Casquete R., Rodríguez A., Hernández A., Martín A., Bartolomé T., Córdoba J.J., Córdoba M.G. (2017). Occurrence of Toxigenic Fungi and Mycotoxins during Smoked Paprika Production. J. Food Prot..

[10] Iqbal Q., Amjad M., Asi M.R. (2011). Assessment of Hot Peppers for Aflatoxin and Mould Proliferation during Storage. J. Food Prot..

[11] Costa J., Rodríguez R., Santos C., Soares C., Lima N., Santos C. (2020). Mycobiota in Chilean chilli *Capsicum annuum* L. used for production of Merkén. Int. J. Food Microbiol..

[12] Frimpong G.K., Adekunle A.A., Ogundipe O.T., Solanki M.K., Sadhasivam S. (2019). Identification and Toxigenic Potential of Fungi Isolated from *Capsicum* Peppers. Microorganisms.

[13] Santos L., Marín S., Mateo E.M., Gil-Serna J., Valle-Algarra F.M., Patiño B., Ramos A.J. (2011). Mycobiota and co-occurrence of mycotoxins in *Capsicum* powder. Int. J. Food Microbiol..

[14] Gambacorta L., Magistà D., Perrone G., Murgolo S., Logrieco A.F., Solfrizzo M. (2018). Co-occurrence of toxigenic moulds, aflatoxins, ochratoxin A, *Fusarium* and *Alternaria* mycotoxins in fresh sweet peppers (*Capsicum annuum*) and their processed products. World Mycotoxin J..

[15] Rapid Alert System for Food and Feed Hazards (RASFF)—European Commission. https://webgate.ec.europa.eu/rasff-window/portal/.

[16] Chilean Ministry of Health. https://www.portaltransparencia.cl/PortalPdT/ingreso-sai-v2?idOrg=undefined.

[17] Ostry V., Malir F., Toman J., Grosse Y. (2017). Mycotoxins as human carcinogens—The IARC Monographs classification. Mycotoxin Res..

[18] Benkerroum N. (2020). Chronic and Acute Toxicities of Aflatoxins: Mechanisms of Action. Int. J. Environ. Res. Public Health.

[19] Costa J., Lima N., Santos C. (2021). An overview on possible links between aflatoxin B1 exposure and gallbladder cancer. Mycotoxin Res..

[20] Imaoka T., Yang J., Wang L., Mcdonald M.G., Afsharinejad Z., Bammler T.K., van Ness K., Yeung C.K., Rettie A.E., Himmelfarb J. (2020). Microphysiological system modeling of ochratoxin A-associated nephrotoxicity. Toxicology.

[21] Chilean Ministry of Health (2013). Decreto 22. https://www.bcn.cl/leychile/navegar?idNorma=1054913.

[22] Ham H., Kim S., Kim M.H., Lee S., Hong S.K., Ryu J.G., Lee T. (2016). Mycobiota of ground red pepper and their aflatoxigenic potential. J. Microbiol..

[23] Kiseleva M., Chalyy Z., Sedova I., Aksenov I. (2020). Stability of Mycotoxins in Individual Stock and Multi-Analyte Standard Solutions. Toxins.

[24] Samson R.A., Hocking A.D., Pitt J.I., King A.D. (1992). Modern methods in food mycology. Developments in Food Science.

[25] International Seed Testing Association-ISTA. https://www.seedtest.org/en/home.html.

[26] Nelson P.E., Toussoun T.A., Marasas W.F.O. (1983). Fusarium Species: An Illustrated Manual for Identification.

[27] Klich M.A. (2002). Identification of Common Aspergillus Species.

[28] Samson R.A., Hockstra E.S., Frisvad J.C., Filtenborg O. (2000). Introduction to Food and Airborne Fungi.

[29] Rodrigues P., Venâncio A., Kozakiewicz Z., Lima N. (2009). A polyphasic approach to the identification of aflatoxigenic and non-aflatoxigenic strains of *Aspergillus* section *Flavi* isolated from Portuguese almonds. Int. J. Food Microbiol..

[30] Schoch C.L., Seifert K.A., Huhndorf S., Robert V., Spouge J.L., Levesque C.A. (2012). Nuclear ribosomal internal transcribed spacer (ITS) region as a universal DNA barcode marker for fungi. Proc. Natl. Acad. Sci. USA.

[31] Visagie C.M., Houbraken J., Frisvad J.C., Hong S.-B., Klaassen C.H.W., Perrone G., Seifert K.A., Varga J., Yaguchi T., Samson R.A. (2014). Identification and nomenclature of the genus *Penicillium*. Stud. Mycol..

[32] Samson R.A., Visagie C.M., Houbraken J., Hong S.-B., Hubka V., Klaassen C.H.W., Perrone G., Seifert K.A., Susca A., Tanney J.B. (2014). Phylogeny, identification and nomenclature of the genus *Aspergillus*. Stud. Mycol..

[33] White T.J., Burns T., Lee S., Taylor J., Gelgard D.H., Sninsky J.J., White T.J. (1990). Amplification and direct sequencing of fungal ribosomal RNA genes for phylogenetics. PCR Protocols: A Guide to Methods and Applications.

[34] Glass L., Donaldson G.C. (1995). Development of primer sets designed for use with the PCR to amplify conserved genes from filamentous ascomycetes. Appl. Environ. Microbiol..

[35] Bragulat M.R., Abarca M.L., Cabanes F.J. (2001). An easy screening method for fungi producing ochratoxin A in pure culture. Int. J. Food Microbiol..

[36] Chuaysrinule C., Maneeboon T., Roopkham C., Mahakarnchanakul W. (2020). Occurrence of aflatoxin- and ochratoxin A-producing *Aspergillus* species in Thai dried chilli. J. Agric. Res..

[37] Araújo C.A.S., Ferreira P.C., Pupin B., Dias L.P., Avalos J., Edwards J., Hallsworth J.E., Rangel D.E.N. (2020). Osmotolerance as a determinant of microbial ecology: A study of phylogenetically diverse fungi. Fungal Biol..

[38] Butinar L., Zalar P., Frisvad J.C., Gunde-Cimerman N. (2005). The genus *Eurotium* members of indigenous fungal community in hypersaline waters of salterns. FEMS Microbiol. Ecol..

[39] Yogendrarajah P., Jacxsens L., Saeger S., Meulenaer B. (2014). Co-occurrence of multiple mycotoxins in dry chilli (*Capsicum annum* L.) samples from the markets of Sri Lanka and Belgium. Food Control.

[40] Ozbey F., Kabak B. (2012). Natural co-occurrence of aflatoxins and ochratoxin A in spices. Food Control.

[41] Tosun A., Ozden S. (2015). Ochratoxin A in red pepper flakes commercialised in Turkey. Food Addit. Contam. B Surveill..

[42] Ikoma T., Tsuchiya Y., Asai T., Okano K., Ito N., Endoh K., Yamamoto M., Nakamura K. (2015). Ochratoxin A Contamination of Red Chili Peppers from Chile, Bolivia and Peru, Countries with a High Incidence of Gallbladder Cancer. Asian Pac. J. Cancer Prev..

[43] Foerster C., Muñoz K., Delgado-rivera L., Rivera A., Cortés S., Müller A., Arriagada G., Ferreccio C., Rios G. (2019). Occurrence of relevant mycotoxins in food commodities consumed in Chile. Mycotoxin Res..

[44] Foerster C., Gisela R., Patricia G., Muñoz K., Cort S. (2021). Assessment of Mycotoxin Exposure in a Rural County of Chile by Urinary Biomarker Determination. Toxin.

[45] Asai T., Tsuchiya Y., Okano K., Piscoya A., Nishi C.Y., Ikoma T., Oyama T., Ikegami K., Yamamoto M. (2012). Aflatoxin Contamination of Red Chili Pepper from Bolivia and Peru, Countries with High Gallbladder Cancer Incidence Rates. Asian Pac. J. Cancer Prev..

[46] Nogueira L., Foerster C., Groopman J., Egner P., Koshiol J., Ferreccio C. (2015). Association of Aflatoxin with Gallbladder Cancer in Chile. JAMA.

[47] Serra I., Yamamoto M., Calvo A., Cavada G., Báez S., Endoh K., Watanabe H., Tajima K. (2012). Association of chili pepper consumption, low socioeconomic status and longstanding gallstones with gallbladder cancer in a Chilean population. Int. J. Cancer.

[48] Tsuchiya Y., Terao M., Okano K., Nakamura K., Oyama M., Ikegami K., Yamamoto M. (2011). Mutagenicity and Mutagens of the Red Chili Pepper as Gallbladder Cancer Risk Factor in Chilean Women. Asian Pac. J. Cancer Prev..

[49] Cabral L.C., Terminiello L., Fernández Pinto V., Fog Nielsen K., Patriarca A. (2016). Natural occurrence of mycotoxins and toxigenic capacity of *Alternaria* strains from mouldy peppers. Int. J. Food Microbiol..

[50] Santos L., Marín S., Sanchis V., Ramos A.J. (2010). Co-occurrence of aflatoxins, ochratoxin A and zearalenone in *Capsicum* powder samples available on the Spanish market. Food Chem..

[51] European Commission Regulation (EC) (2012). No 594/2012 of, 5 July 2012 amending Regulation (EC) 1881/2006 as regard the maximum levels of the contaminants ochratoxin A, non-dioxin-like PCBs and melamine in foodstuffs. Off. J. Eur. Union.

[52] European Commission Regulation (EU) (2015). No 2015/1137, of 13 July 2015 amending Regulation (EC) No 1881/2006 as regards the maximum level of Ochratoxin A in *Capsicum* spp. spices. Off. J. Eur. Union.

[53] Food and Agriculture Organization of the United Nations-FAO. http://www.fao.org/fao-whocodexalimentarius.

[54] Buitimea-Cantúa G.V., Buitimea-Cantúa N.E., Rocha-Pizaña M.D.R., Hernández-Morales A., Magaña-Barajas E., Molina-Torres J. (2020). Inhibitory effect of *Capsicum chinense* and *Piper nigrum* fruits, capsaicin and piperine on aflatoxins production in *Aspergillus parasiticus* by downregulating the expression of afl D, afl M, afl R, and afl S genes of aflatoxins biosynthetic pathway. J. Environ. Sci. Health-B.

[55] Tewksbury J.J., Reagan K.M., Machnicki N.J., Carlo A., Haak D.C., Peñaloza A., Levey D.J. (2008). Evolutionary ecology of pungency in wild chilies. Proc. Natl. Acad. Sci. USA.

[56] Coton M., Auffret A., Poirier E., Debaets S., Coton E. (2019). Production and migration of ochratoxin A and citrinin in Comté cheese by an isolate of *Penicillium verrucosum* selected among *Penicillium* spp. mycotoxin producers in YES medium. Food Microbiol..

[57] Delgado J., Cruz Cabral L., Rodríguez M., Rodríguez A. (2018). Influence of ochratoxin A on adaptation of *Penicillium nordicum* on a NaCl-rich dry-cured ham-based medium. Int. J. Food Microbiol..

[58] Ferrara M., Magistà D., Epifani F., Cervellieri S., Lippolis V., Gallo A., Perrone G., Susca A. (2016). Study of gene expression and OTA production by *Penicillium nordicum* during a small-scale seasoning process of salami. Int. J. Food Microbiol..

[59] Rodríguez A., Medina Á., Córdoba J.J., Magan N. (2014). The influence of salt (NaCl) on ochratoxin A biosynthetic genes, growth and ochratoxin A production by three strains of *Penicillium nordicum* on a dry-cured ham-based medium. Int. J. Food Microbiol..

[60] Schmidt-Heydt M., Graf E., Stoll D., Geisen R. (2012). The biosynthesis of ochratoxin A by *Penicillium* as one mechanism for adaptation to NaCl rich foods. Food Microbiol..

[61] Masih A., Singh P.K., Kathuria S., Agarwal K., Meis J.F., Chowdhary A. (2016). Identification by molecular methods and matrix-assisted laser desorption ionization-time of flight mass spectrometry and antifungal susceptibility profiles of clinically significant rare *Aspergillus* species in a referral chest hospital in Delhi, India. J. Clin. Microbiol..

[62] Sabz G., Gharaghani M., Mirhendi H., Ahmadi B., Gatee M.A., Sisakht M.T., Hemati A., Mohammadi R., Taghavi J., Nouripour-Sisakht S. (2019). Clinical and microbial epidemiology of otomycosis in the city of Yasuj, southwest Iran, revealing *Aspergillus tubingensis* as the dominant causative agent. J. Med. Microbiol..

[63] Santos R.A.C., Steenwyk J.L., Rivero-Menendez O., Mead M.E., Silva L.P., Bastos R.W., Alastruey-Izquierdo A., Goldman G.H., Rokas A. (2020). Genomic and Phenotypic Heterogeneity of Clinical Isolates of the Human Pathogens *Aspergillus fumigatus*, *Aspergillus lentulus*, and *Aspergillus fumigatiaffinis*. Front. Genet..

[64] Meteochile. http://www.meteochile.gob.cl/PortalDMC-web/index.xhtml.

[65] Turner P.C., Sylla A., Gong Y.Y., Diallo M.S., Sutcliffe A.E., Hall A.J., Wild C.P. (2005). Reduction in exposure to carcinogenic aflatoxins by postharvest intervention measures in west Africa: A community-based intervention study. Lancet.

